# Identification of differentially expressed long non-coding RNAs in human ovarian cancer cells with different metastatic potentials

**DOI:** 10.7497/j.issn.2095-3941.2013.03.003

**Published:** 2013-09

**Authors:** Shi-Ping Liu, Jia-Xin Yang, Dong-Yan Cao, Keng Shen

**Affiliations:** Department of Obstetrics and Gynecology, Peking Union Medical College Hospital, Peking Union Medical College, Chinese Academy of Medical Sciences, Beijing 100730, China

**Keywords:** Neoplasm metastasis, ovarian neoplasms, RNA, long untranslated

## Abstract

**Objective:**

To identify differentially expressed long non-coding RNAs (lncRNAs) involved in the metastasis of epithelial ovarian cancer.

**Methods:**

An *in vitro* invasion assay was performed to validate the invasive capability of SKOV3 and SKOV3.ip1 cell lines. Total RNA was then extracted, and microarray analysis was performed. Moreover, nine lncRNAs were selected for validation using RT-qPCR.

**Results:**

Compared with the SKOV3 cells, the SKOV3.ip1 cells significantly improved in the *in vitro* invasive activity. Of the 4,956 lncRNAs detected in the microarray, 583 and 578 lncRNAs were upregulated and downregulated, respectively, in SKOV3.ip1 cells, compared with the parental SKOV3 cells. Seven of the analyzed lncRNAs (MALAT1, H19, UCA1, CCAT1, LOC645249, LOC100128881, and LOC100292680) confirmed the deregulation found by microarray analysis.

**Conclusion:**

LncRNAs clusters were differentially expressed in ovarian cancer cells with varying metastatic potentials. This result indicates that some lncRNAs might exert a partial or key role in epithelial ovarian cancer metastasis. Further studies should be conducted to determine the roles of these lncRNAs in ovarian cancer metastasis.

## Introduction

Epithelial ovarian cancer is the leading cause of death from gynecologic cancer in developed regions worldwide and second to cervical cancer in developing countries^1^^,^^2^. Although new therapeutic drugs have been developed to treat ovarian cancer patients, the mortality rate among women diagnosed with metastatic disease remains high, with only 27% of them surviving more than 5 years post-diagnosis^3^. Therefore, the identification of genes and regulatory mechanisms involved in metastasis that could enhance the understanding of cancer progression and result in the development of new therapeutics has attracted much interest.

Long non-coding RNAs (lncRNAs) are non-coding RNAs that are longer than 200 nucleotides. Recent studies have demonstrated that lncRNAs play important roles in carcinogenesis and cancer metastasis. Aberrant lncRNA profiles have been noted in multiple cancers^4^^-^^6^, but the lncRNAs involved in ovarian cancer metastasis have not been identified. In this study, we presented the lncRNA expression profiles in paired ovarian cancer cell lines with different metastatic potentials and selected nine differentially expressed lncRNAs for validation using RT-qPCR.

## Materials and methods

### Cell lines and cell culture

The human ovarian cancer cell lines SKOV3 and SKOV3.ip1 were obtained from the American Type Culture Collection and M.D. Anderson Cancer Center, respectively. The SKOV3.ip1 cell line was derived from ascites from a nude mouse injected i.p. with SKOV3 cells and demonstrated a higher metastatic potential compared with the parental cell line^7^. Both cell lines were maintained in Dulbecco’s modified Eagle’s medium supplemented with 10% fetal bovine serum and cultured in a humidified incubator (5% CO_2_) at 37 °C.

### Validation of invasive capability of ovarian cancer cells

*In vitro* invasion assay was performed using 24-well transwell units with polycarbonate filters (pore size: 8 μm) coated on the upper side with reconstituted basement membrane matrix (BD Biosciences, USA). The cells were harvested, and 4×10^4^ cells in 100 μL of serum-free medium were placed in the upper part of the transwell unit and were allowed to invade the membrane for 72 h at 37 °C. Successfully penetrating cells were fixed, stained, and quantified at optical density of 570 nm after extraction. The results are reported as the averages of three individual experiments containing three replicates per condition.

### RNA preparation

Total RNA was extracted from the cell samples using the TRIzol reagent (Invitrogen) according to the manufacturer’s instructions. The RNA integrity was analyzed using the Agilent 2100 Bioanalyzer (Agilent Technologies).

### Microarray

Microarray analysis was performed by a commercial company (Oebiotech, PRC), using SurePrint G3 Human Gene Expression 8×60K v2 (Agilent Technologies) that is designed to contain approximately 12,000 lncRNAs. Briefly, samples were used to synthesize cDNA, and labeled cRNA was then synthesized and hybridized to the microarray. After hybridization and washing, processed slides were scanned with the Agilent Microarray Scanner (Agilent Technologies), and the acquired array images were analyzed using Agilent Feature Extraction Software (Agilent Technologies), which performs background subtractions. Quantile normalization and subsequent data processing were performed using the GeneSpringGX v. 11.0 software package (Agilent Technologies). A threshold of fold change >2 was used to screen upregulated or downregulated lncRNAs.

### Real-time quantitative PCR validation

To validate the microarray data, we selected nine differentially expressed lncRNAs (MALAT1, H19, XIST, UCA1, CCAT1, LOC645249, LOC100128881, LOC728228, and LOC100292680). MALAT1, H19, XIST, UCA1, and CCAT1 were selected because they had been associated with cancer. The four remaining lncRNAs were selected randomly from all aberrantly expressed lncRNAs.

Quantification was performed with a two-step reaction process, i.e., through reverse transcription (RT) and PCR. Each RT reaction consisted of 0.5 μg RNA, 2 μL of PrimerScript Buffer, 0.5 μL of oligo dT, 0.5 μL of random 6 mers, and 0.5 μL of PrimerScript RT Enzyme Mix I (TaKaRa, Japan), in a total volume of 10 μL. Reactions were performed in a GeneAmp® PCR System 9700 (Applied Biosystems, USA) for 15 min at 37 °C, followed by heat inactivation of RT for 5 s at 85 °C. The 10 μL RT reaction mix was then diluted ×10 in nuclease-free water and held at –20 °C.

Real-time PCR was performed using LightCycler® 480 II Real-time PCR Instrument (Roche, Swiss) with 10 μL PCR reaction mixture that included 1 μL of cDNA, 5 μL of 2× LightCycler® 480 SYBR Green I Master (Roche, Swiss), 0.2 μL of forward primer, 0.2 μL of reverse primer, and 3.6 μL of nuclease-free water. Reactions were incubated in a 384-well optical plate (Roche, Swiss) at 95 °C for 10 min, followed by 40 cycles at 95 °C for 10 s, 60 °C for 30 s. At the end of the PCR cycles, melting curve analysis was performed to validate the specific generation of the expected PCR product. Three independent experiments were performed with each sample run in triplicate. The primers were synthesized by Generay Biotech (Generay, PRC) and are listed in **Table 1**. The expression levels of lncRNAs were normalized with respect to GAPDH and were calculated using the 2^–ΔΔCt^ method.

**Table 1:** Primers used in RT-qPCR

Gene symbol	Forward primer	Reverse primer
MALAT1	CAGCAGCAGACAGGATTC	TTCCTTCACCAAATCGCAC
H19	TTTCATCCTTCTGTCTCTTTGT	CAACCAGTGCAAATGACTTAG
XIST	TGACCTTGTTAAGCAAGCG	ATGGACCACTGTTTGATAGAC
UCA1	TCGGGTAACTCTTACGGT	GGTCCATTGAGGCTGTAG
CCAT1	AGAAACACTATCACCTACGC	CTTAACAGGGCATTGCTAATCT
LOC645249	TGGGAGGAGGAGAGGGTTA	GTGTGTGTATTTGTGCTTCTT
LOC100128881	CGGCTCTACCACTGTTACTTA	GTGCTCCATGCCAAGCTA
LOC728228	CCTTCATGCCTGTCCTTT	CCTCACCAACAACCGCTA
LOC100292680	GAGAGGGTTGAAGTTCCG	TGTTCTAGGTCGCTCGTC
GAPDH	TGTTGCCATCAATGACCCCTT	CTCCACGACGTACTCAGCG

### Statistical analysis

Student’s *t*-test was performed to evaluate any significant difference. A value of *P*<0.05 was considered statistically significant.

## Results

### Increased invasive capability of SKOV3.ip1 cells

**Figure 1:**
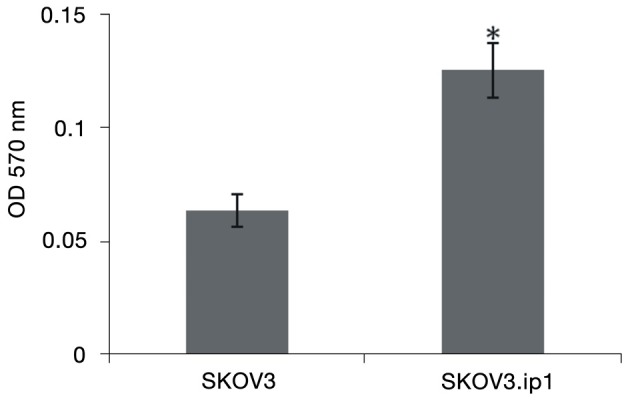
SKOV3.ip1 cells are more invasive than SKOV3 cells (*P<0.01).

The abilities of the two cell lines to penetrate a uniform layer of extracellular matrix-resembling material were tested using Transwell invasive assay to compare their invasive capabilities. Cells were seeded on top of a porous membrane. Fetal bovine serum was used as a chemoattractant in the lower compartment. The results showed a marked increase in the invasive ability of the SKOV3.ip1 cell line compared with that of the SKOV3 cell line (**Figure 1**).

### Differentially expressed metastasis-related lncRNAs

A total of 4,956 lncRNAs were detected in the microarray. Data analysis showed that, compared with the parental SKOV3 cells, more than a 2-fold deregulation was found in 1,161 lncRNAs in SKOV3.ip1 cells, of which 583 were lncRNAs were upregulated and 578 downregulated. The expression levels of nine lncRNAs were validated using RT-qPCR, and seven confirmed the deregulation found by microarray analysis (**Table 2**).

**Table 2:** Deregulated genes in SKOV3.ip1 compared to SKOV3 cells

Gene symbol	Microarray fold change	RT-qPCR fold change	*P*
MALAT1	–3.2	–2.1	0.009
H19	–18.4	–24.4	0.028
UCA1	9.0	3.5	0.003
CCAT1	2.6	6.7	0.007
LOC645249	8.5	7.6	0.000
LOC100128881	7.9	5.6	0.032
LOC100292680	–6.0	–6.9	0.002

## Discussion

Cancer metastasis is the leading cause of death in cancer patients^8^. In the current study, we primarily identified the lncRNAs involved in the metastasis of epithelial ovarian cancer. We analyzed paired high and low metastatic ovarian cancer cells by microarray assay and selected nine lncRNAs for validation by RT-qPCR. Interestingly, we found that 1,161 of the 4,956 detected lncRNAs were differentially expressed between the two cell lines. Among these 1,161 lncRNAs, 583 and 578 were found to be upregulated and downregulated, respectively. Most of these lncRNAs have not been functionally characterized. These lncRNAs may be identified as novel biomarkers and therapeutic targets.

Nine lncRNAs were selected for validation on the basis of relevant literature and the microarray data. The qPCR results of seven lncRNAs (MALAT1, H19, UCA1, CCAT1, LOC645249, LOC100128881, and LOC100292680) were consistent with the deregulation found by microarray analysis, reflecting the reliability of the microarray data to some extent. Of the seven lncRNAs, H19 was markedly downregulated in the SKOV3.ip1 cells. Similarly, Zhang *et al.*^9^ reported that H19 suppressed hepatocellular carcinoma metastasis and the expression of markers of epithelial-to-mesenchymal transition by altering the miR-200 pathway. However, Yang *et al.*^10^ reported that H19 upregulation increased cell proliferation in gastric cancer cells. Furthermore, another study has found that H19 was remarkably upregulated in bladder cancer tissues, and upregulated H19 promoted cancer cell migration both *in vitro* and *in vivo* by associating with EZH2 and inhibiting E-cadherin expression^11^. Another lncRNA, MALAT1, is known to promote cancer metastasis in several cancer types, including lung, bladder, and cervical cancers^12^^-^^14^, and its overexpression has been reported to predict tumor recurrence of hepatocellular carcinoma after liver transplantation^15^. However, in the current study, it was downregulated in the cell culture with high metastatic potential cells. Thus, the function of one lncRNA may vary with different cancer types and context. H19 and MALAT1 may play a suppressive role in ovarian cancer metastasis. Published data on UCA1 and CCAT1 are limited. One study has suggested that UCA1 might contribute to bladder cancer progression^16^, and CCAT1 has been reported to be overexpressed in colon cancer^17^^,^^18^. The remaining three lncRNAs are uncharacterized, and their biological functions remain unclear.

To our knowledge, this study is the first to describe the lncRNA profiles in paired high and low metastatic ovarian cancer cells. A number of differentially expressed lncRNAs were identified, and these lncRNAS may play a partial or key role in the metastasis of epithelial ovarian cancer. Further studies are necessary to determine the roles of these lncRNAs in ovarian cancer metastasis.
